# Genome-wide association study on soybean canopy wilting under drought stress conditions in a rainout-shelter greenhouse

**DOI:** 10.3389/fpls.2026.1840313

**Published:** 2026-06-09

**Authors:** Hyeonjun Cho, Dongho Lee, Sangyeab Lee, Seungmin Choi, Deokmoon Lee, Yunseok Lee, Jiyoung Park, Kihwan Kim, Hyun Jo, Jong Tae Song, Jeong-Dong Lee

**Affiliations:** 1 Department of Applied Biosciences, Kyungpook National University, Daegu, Republic of Korea; 2 Crop, Soil and Environmental Sciences, University of Arkansas, Fayetteville, AR, United States; 3 Upland Field Machinery Research Center, Kyungpook National University, Daegu, Republic of Korea; 4 Department of Integrative Biology, Kyungpook National University, Daegu, Republic of Korea

**Keywords:** drought, gene, GWAS, LD analysis, soybean

## Abstract

Drought is one of the most important abiotic stresses affecting plant growth and productivity worldwide. Soybean [*Glycine max* (L.) Merr.] is particularly sensitive to drought stress, which can cause yield losses of up to 40%. This study aimed to identify genomic regions associated with canopy wilting, a key indicator of drought response in soybean, using genome-wide association analysis. A total of 286 soybean accessions obtained from the National Agrobiodiversity Center, Republic of Korea, were evaluated for canopy wilting under drought conditions in a rainout-shelter greenhouse over three consecutive years. The accessions were genotyped using the Axiom^®^ 180K SoyaSNP array, and a total of 68,375 high-quality single-nucleotide polymorphisms (SNPs) were used for genome-wide association analysis with the Bayesian-information and Linkage-disequilibrium Iteratively Nested Keyway (BLINK) model. Six significant SNPs were identified on chromosomes 5, 9, 12, 13, and 14, suggesting a polygenic genetic architecture of canopy wilting. All significant SNPs were located within ±1 Mb of genomic regions previously reported to be associated with canopy wilting-related traits in soybean. Linkage disequilibrium (LD) analysis identified an LD block encompassing the significant SNPs, within which 36 candidate genes were detected. Several of these genes were related to receptor-like kinases, RNA-processing proteins, and stress-responsive metabolic pathways, suggesting that multiple regulatory mechanisms contribute to canopy wilting responses in soybean. These findings enhance our understanding of the genetic architecture underlying drought tolerance in soybean and provide valuable genetic resources to facilitate marker-assisted selection for the development of drought-tolerant cultivars.

## Introduction

Soybean [*Glycine max* (L.) Merr.] is one of the most important crops because of its high nutritional value and oil content, making it a versatile commodity for human food, animal feed, and various industrial applications (Liu, 1997; Medic et al., 2014). Reflecting a steady expansion in global soybean-producing area over recent decades, global soybean production has continued to grow from 378 million metric tons in 2022/2023 to 397 million metric tons in 2023/2024, projecting to 421 million metric tons in 2024/2025 (www.soystats.com). However, ongoing global warming has intensified climate-related constraints on crop productivity, leading to a continuous increase in drought-affected arable areas (Dai, 2013).

Drought stress can typically reduce soybean yield by up to 40% (Saxena, 2003; Manavalan et al., 2009; Westcott and Jewison, 2013). However, the yield reduction can be exacerbated when drought stress is imposed during the flowering (R1–R2) and seed-filling (R5–R6) stages, with yield reductions ranging from 73% to 82% (Wei et al., 2018). These reductions are largely associated with physiological limitations under water deficit, including reduced transpiration, stomatal closure, and decreased photosynthetic carbon assimilation, which collectively impair reproductive processes requiring adequate water availability and ultimately lead to reduced seed yield. At the canopy level, drought stress manifests as reduced leaf expansion, decreased chlorophyll content, and lower stomatal conductance, which collectively limit transpiration and simultaneously constrain photosynthetic carbon assimilation (Ohashi et al., 2006; Makbul et al., 2011; Silvente et al., 2012). In addition, plants undergo osmotic adjustment through the accumulation of compatible solutes, including soluble sugars, sugar alcohols, and amino acids, which contribute to the maintenance of cell turgor under reduced water availability (Jones and Turner, 1978; Manavalan et al., 2009). Additionally, drought stress induces modifications in root system architecture, including changes in root length, density, and spatial distribution, which enhance soil water acquisition and contribute to drought adaptation in soybean (Benjamin and Nielsen, 2006; Fenta et al., 2014). The capacity of plants to mitigate the adverse effects of water limitation through adaptive traits is referred to as drought tolerance (Basu et al., 2016). One of the key traits associated with improved drought tolerance is slow canopy wilting (SW), which has been widely used as a reliable indicator for evaluating drought tolerance in soybean under field conditions (Charlson et al., 2009).

Canopy wilting, defined as the loss of turgor and leaf drooping, is a common physiological response of soybean plants under drought conditions. However, certain exotic soybean accessions exhibit a slower or delayed wilting response, which is often associated with advantageous traits such as improved soil water uptake, enhanced water conservation prior to stress, or greater water-use efficiency. Given that canopy wilting is a key physiological trait associated with drought tolerance, understanding its genetic basis is essential for improving drought adaptation in soybean. As a complex trait influenced by multiple genetic and environmental factors, dissecting its underlying genetic architecture is critical for identifying loci associated with drought response. Genetic approaches such as quantitative trait locus (QTL) mapping and genome-wide association studies (GWAS) provide effective tools for dissecting the genetic architecture of complex traits such as canopy wilting.

Previous studies implemented QTL analysis to identify genomic regions associated with SW. Using 92 recombinant inbred lines (RILs) derived from cultivars KS4895 and Jackson, four QTLs associated with SW were detected on chromosomes (Chrs.) 8, 13, 14, and 17 (Charlson et al., 2009). Another RIL population derived from PI 416937 and Benning was used to identify seven SW-associated QTLs on Chrs. 2, 4, 5, 12, 14, 17, and 19 (Abdel-Haleem et al., 2012). More recently, two SW-associated QTLs on Chrs. 6 and 10 were identified using a RIL population derived from a cross between Magellan and PI 567731 (Ye et al., 2020). In addition, GWAS have been widely employed to explore the genetic architecture of slow wilting using high-density marker datasets and diverse accession panels. Several GWAS have investigated canopy wilting in soybean using large and diverse panels under field-based conditions. Kaler et al. (2017) evaluated canopy wilting in open-field trials across multiple environments and identified 61 significant single-nucleotide polymorphisms (SNPs) and 51 putative genomic regions associated with slow wilting. Similarly, Steketee et al. (2020) assessed canopy wilting in a panel of 162 soybean accessions under rain-fed field conditions with minimal or no rainfall and identified 44 significant SNPs. More recently, Chamarthi et al. (2021) evaluated a panel of 200 accessions with three replications under both irrigated and rain-fed treatments and identified 188 significant SNPs corresponding to 152 loci.

Despite these efforts, GWAS of canopy wilting have mainly been conducted under field-based environments, where environmental heterogeneity and inconsistent drought stress can limit the accurate detection of genetic effects. To address this limitation, controlled drought systems such as rainout-shelter greenhouses provide a more uniform environment, enabling more reliable phenotypic evaluation and improved detection of genetic loci associated with drought-responsive traits. However, relatively few studies have examined the genetic architecture of slow wilting under such controlled conditions. Therefore, the objective of this study was to identify genomic regions associated with slow wilting through GWAS using 286 diverse soybean accessions evaluated under rainout-shelter greenhouse drought stress conditions.

## Materials and methods

### Plant materials and rainout-shelter greenhouse experimental design

A panel of 286 soybean accessions was obtained from the National Agrobiodiversity Center (Rural Development Administration, Republic of Korea). The association panel comprised 286 soybean accessions originating from 48 countries. Korean accessions accounted for the largest proportion (*n* = 126), followed by the United States (*n* = 30), China (*n* = 30), and Japan (*n* = 10). Days to maturity among the 286 soybean accessions ranged from 84 to 157 days under field conditions. A wide distribution of maturity periods was observed across the panel. A total of 208 accessions (approximately 75%) required more than 116 days to reach maturity, indicating that late-maturing genotypes predominated in the population (**Supplementary Table 1**). Information on flowering and maturity traits was obtained from the National Agrobiodiversity center. Population structure was accounted for using principal component analysis (PCA), and the first two principal components were included as covariates in the GWAS model (Huang et al., 2019). The diverse panel was grown in a rainout-shelter greenhouse (**Supplementary Figure 1**) to evaluate drought tolerance over three consecutive years (2021–2023) at Kyungpook National University, Gunwi, Republic of Korea (36°07′N, 128°38′E). Each genotype was planted in a hill plot with 15 cm between hills and 50 cm between rows. The hill plots were managed under a custom irrigation system consisting of a water pump, PVC pipes, and dripper lines. Water was pumped from a storage tank through PVC header pipes and then delivered uniformly to each hill plot. From the headers, water was distributed directly to the plant rows via NetafimTM Techline dripper lines (emitter spacing of 15 cm, flow rate of 1.49 L/h per emitter; Netafim, Fresno, California). The dripper emitters were standardized to ensure that all plants received the same amount of water. Irrigation was discontinued once the plants reached the V2–V3 growth stages. Soil moisture conditions were monitored using soil water potential (pF) measurements obtained with TEROS 21 soil water potential sensors (METER Group, USA) throughout the drought period. Detailed soil moisture data were recorded for the 2022 growing season (**Supplementary Table 6**), confirming a progressive decline in soil water availability during the experimental period. The experiment was conducted using a randomized complete block design with two replications in 2021, five replications in 2022, and two replications in 2023.

### Screening of canopy wilting and data processing

Drought stress was imposed at the early vegetative stage (V3–V5), and phenotypic evaluations were conducted during the mid-to-late growing season, when the majority of accessions had transitioned into the early reproductive phase, while a subset of later-maturing accessions remained at late vegetative or transitional stages, depending on maturity group (**Supplementary Table 2**). Canopy wilting was visually scored on a scale of 1 to 5, where a wilting score of 1 represents no wilting; 2 represents a few upper leaves with slight wilting and rolling; 3 represents 50% of leaves showing wilting; 4 represents 75% of leaves showing wilting, with advanced loss of petiole turgidity; and 5 represents all leaves showing wilting or dead plants (**Supplementary Figure 1**). Adjusted means across environments for each genotype were calculated using the *emmeans* function in the *emmeans* package (Lenth et al., 2024) in R software (v4.5.2; R core team 2025) based on a mixed linear model with genotype treated as a fixed effect and environment, genotype × environment interaction, and replication nested within environment as random effects (Equation 1). All experiments were conducted at the same location, and therefore, the environmental variation in this study reflects differences among years. The model was fitted using the *lmer* function in the *lmerTest* package (Bates et al., 2014; Kuznetsova et al., 2017).

(1) $$Wilting_{ijk}=\;\mu +\;Gen_{i}+{\left(Gen\times Env\right)}_{ij}+{\left(Rep|Env\right)}_{kj}+Env_{j}+\;\epsilon_{ijk}$$

where $Wilting_{ijk}$ represents the canopy wilting score for the *i*th genotype, in the *j*th environment, and the *k*th replication; μ represents the overall mean of the wilting score; $Gen_{i}$ represents the fixed effect for the *i*th genotype; ${\left(Gen\times Env\right)}_{ij}$ represents the random effect for the interaction between the *i*th genotype and the *j*th environment; ${\left(Rep|Env\right)}_{kj}$ represents the random effect for the *k*th replications nested within the *j*th environment; $Env_{i}$ represents the random effect for the *j*th environment; and ${\text{ϵ}}_{ijk}$ represents the residual term error.

### Genotypic data processing and kinship analysis

The diverse panel of 286 soybean accessions was genotyped using the Affymetrix Axiom^®^ 180k SoyaSNP array (Lee et al., 2015) based on the Williams 82 reference genome version 2 (Wm82.a2.v1), of which the genotypic data are publicly available in Soybase (https://www.soybase.org/, accessed on 28 January 2026). Missing SNPs were imputed using BEAGLE version 5.5 (Browning et al., 2018). Afterwards, the imputed genotypic data were filtered based on minor allele frequency less than 0.1 using TASSEL version 5.0 (Bradbury et al., 2007). A total of 68,375 SNPs were obtained and used for further analyses. The number of SNPs mapped across the genome ranged from 2,204 (Chr. 12) to 4,551 (Chr. 18), with an average of 3,419 (**Supplementary Table 3**; **Supplementary Figure 2**). The genetic relatedness and population structure of the panel were evaluated using kinship analysis and PCA in R software.

### Genome-wide association study

The marker–trait associations were detected using the adjusted mean values and filtered SNP markers via the Bayesian-information and Linkage-disequilibrium Iteratively Nested Keyway (BLINK) model (Huang et al., 2019), implemented in the R package “Genome association and prediction integrated tool (*GAPIT*)” (Lipka et al., 2012). The BLINK model is the latest GWAS model in *GAPIT* that improves both statistical and computational power by using a multi-locus approach that accounts for linkage disequilibrium (LD) and Bayesian Information Content. In addition, BLINK incorporates associated markers as covariates in an iterative manner, reducing false positives and improving the detection of true marker–trait associations compared to traditional mixed linear models (MLMs). BLINK is also an extension of the FarmCPU method, designed to enhance statistical power and computational efficiency. To reduce the type II error, a significant threshold of *p*-value = 1.0 × 10^−4^ was used based on the false discovery rate (FDR) at a significant level of 0.01.

### Linkage disequilibrium block and potential candidate genes

The LD blocks harboring significant SNPs were defined using the Haploview software (Barrett et al., 2005). Pairwise LD was estimated and used to identify LD blocks on the default confidence interval-based method. Pairwise comparisons between markers separated by more than 200 kb were excluded (Gabriel et al., 2002). All genes located in the estimated LD blocks were considered potential candidate genes. The gene ID, physical positions, and functional annotations were obtained from Soybase (soybase.org) based on Wm82.a2.v1.

### Soybean expression atlas analysis

All candidate genes identified in this study were further analyzed using a comprehensive RNA-seq dataset from “SoybeanExpressionAtlas” (Almeida-Silva et al., 2023) to examine gene expression patterns across eight soybean tissues: nodule, root, radicle, petiole, hypocotyl, seedling, shoot, and leaf. Only the candidate genes available in the Williams 82 reference genome version 4 (Wm82.a4.v1) were used in this analysis, of which 33 out of 36 candidate genes were included. Raw expression values were normalized to transcripts per million (TPM), and mean TPM values were calculated for each gene across tissues. For visualization, averaged TPM values were log_2_-transformed [log_2_(TPM + 1)] and subsequently standardized by gene using *z*-score normalization.

### Statistical analysis

Analysis of variance (ANOVA) was conducted using the *aov* function in R software. The descriptive statistics, mean, standard deviation, coefficient of variation (CV), and Student’s *t*-test were also analyzed in R software. The phenotypic correlations across environments were evaluated using Spearman’s rank correlation coefficient.

## Results

### Screening of canopy wilting

**Figure 1:**
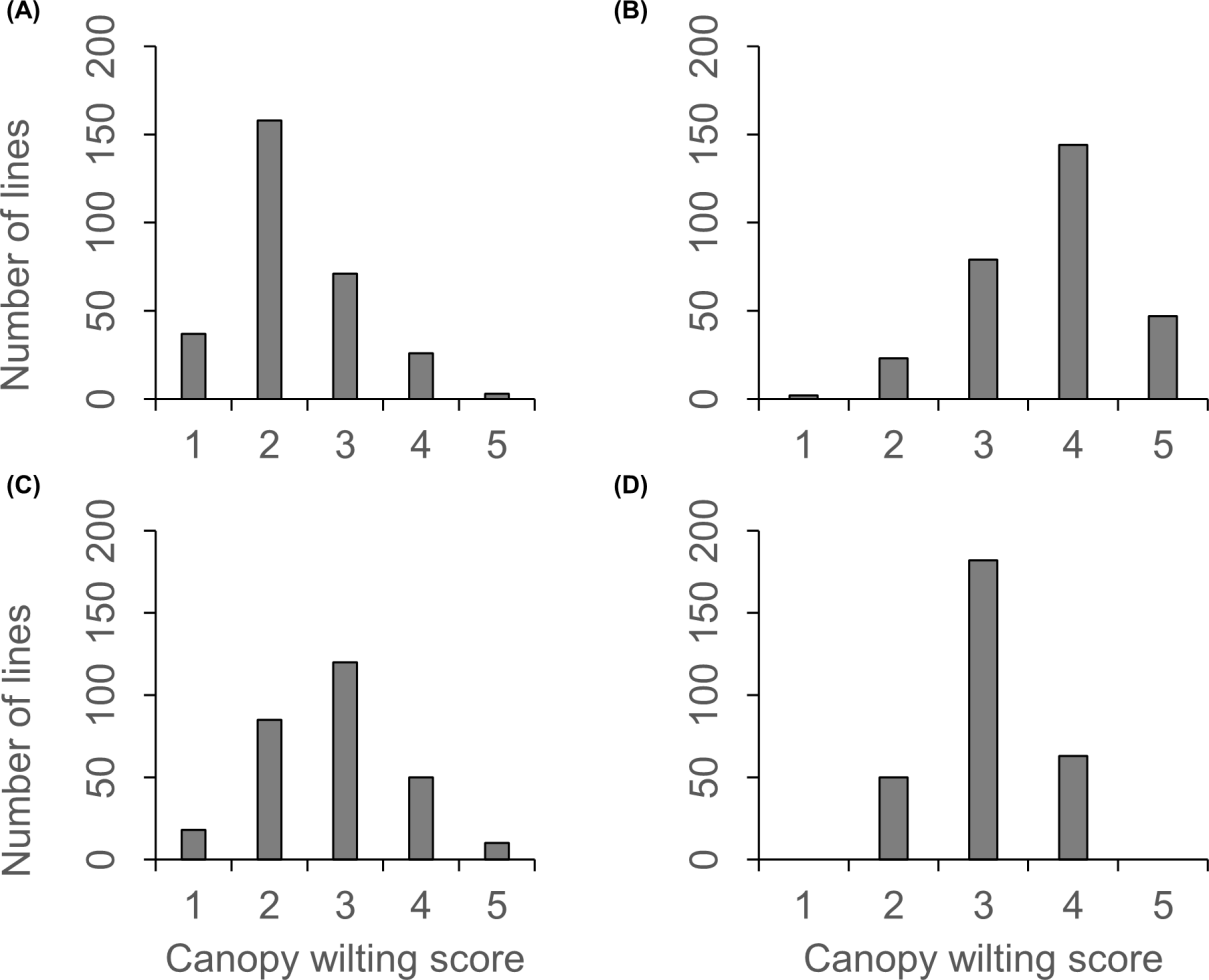
Phenotypic distribution of the canopy wilting score in 286 soybeans in 2021 **(A)**, 2022 **(B)**, 2023 **(C)**, and combined data **(D)**. The canopy wilting score was measured on a scale of 1 to 5, where a wilting score of 1 represented healthy without wilting; 2 represented a few upper leaves with slight wilting and rolling; 3 represented half of all leaves wilting; 4 represented 75% of all leaves wilting, with advanced loss of petiole turgidity; and 5 represented all leaves wilted or dead plants.

The canopy wilting of the 286 soybean accessions was visually evaluated for three consecutive years (2021, 2022, and 2023), which were considered three environments (**Table 1**). The canopy wilting scores ranged from 1.0 to 5.0, with a mean of 2.5 in 2021. The canopy wilting scores in 2022 ranged from 1.8 to 5.0, with a mean of 4.1, whereas in 2023, the scores ranged from 1.0 to 5.0, with a mean of 3.0. The average canopy wilting scores across the environments were 3.2, with a range of 2.1 to 4.8. For each environment, the CV was 33.9% in 2021, 18.3% in 2022, and 28.7% in 2023, whereas the average canopy wilting scores across the environments showed a CV of 16.2%. The phenotypic distribution of canopy wilting scores showed different skewed trends across the environments, showing a right-skewed distribution in 2021, a left-skewed distribution in 2022, and a normal distribution in 2023 and averaged scores (**Figure 1**). The correlation test between environments demonstrated a significant environmental effect, of which the ANOVA results also revealed the significant effects of “genotype” and “environment”, but no significant effect of “genotype × environment” (**Supplementary Figure 3**; **Table 2**). To account for the significant variability in canopy wilting scores across environments, adjusted means were calculated to minimize environmental noise and better estimate the underlying genetic effects associated with canopy wilting.

**Table 1:** Descriptive statistics of canopy wilting scores^2^ across three environments.

Year	Range	Mean	Standard deviation	Coefficient of variance (%)
2021	1.0-5.0	2.5	0.8	33.9
2022	1.8-5.0	4.1	0.8	18.3
2023	1.0-5.0	3.0	0.9	28.7
Combined^1^	2.1-4.8	3.2	0.5	16.2

^1^ Combined values represent accession-wise mean canopy wilting scores averaged across 3 years (2021–2023).

^2^ 1 represented healthy without wilting; 2 represented a few upper leaves with slight wilting and rolling; 3 represented half of all leaves wilting; 4 represented 75% of all leaves wilting, with advanced loss of petiole turgidity; and 5 represented all leaves wilted or dead plants.

**Table 2:** Analysis of variance for canopy wilting scores of 286 cultivated soybean accessions across 3 years.

Source of variation	Degree of freedom (df)	Sum of square (SS)	Mean square (MS)	*F*-value	*p*-value
Genotype (G)	285	748.0	2.6	1.6	***
Year (Y)	2	1,187.3	593.7	372.6	***
G × Y	558	863.4	1.5	1.0	ns
Error	1,690	2,692.7	1.6		

***, **, and * indicate significance at *p* < 0.001, *p* < 0.01, and *p* < 0.05, respectively; ns indicates non-significance.

Differences in canopy wilting responses among years were closely associated with variation in environmental conditions (**Supplementary Table 5**). The mean wilting score was highest in 2022 (4.1), followed by 2023 (3.0) and 2021 (2.5), indicating more severe drought stress in 2022. This pattern was consistent with environmental data, where 2022 exhibited higher mean temperature (25.6 °C), the greatest number of high-temperature observations (≥35 °C; 23 observations), and the lowest precipitation (178.7 mm), suggesting intensified drought and heat stress conditions. In contrast, 2021 showed lower temperature (22.6 °C) and minimal heat exposure (2 observations), corresponding to reduced wilting severity. Although 2023 had similarly high temperatures (25.8 °C), substantially higher precipitation (611.8 mm) likely mitigated drought stress, resulting in intermediate wilting scores. The CV further supported these trends, with the highest variability observed in 2021 (33.9%) and the lowest in 2022 (18.3%), suggesting that moderate stress conditions allowed greater differentiation among genotypes, whereas severe stress resulted in uniformly high wilting across accessions.

### Assessment of genetic diversity among 286 soybean accessions

**Figure 2:**
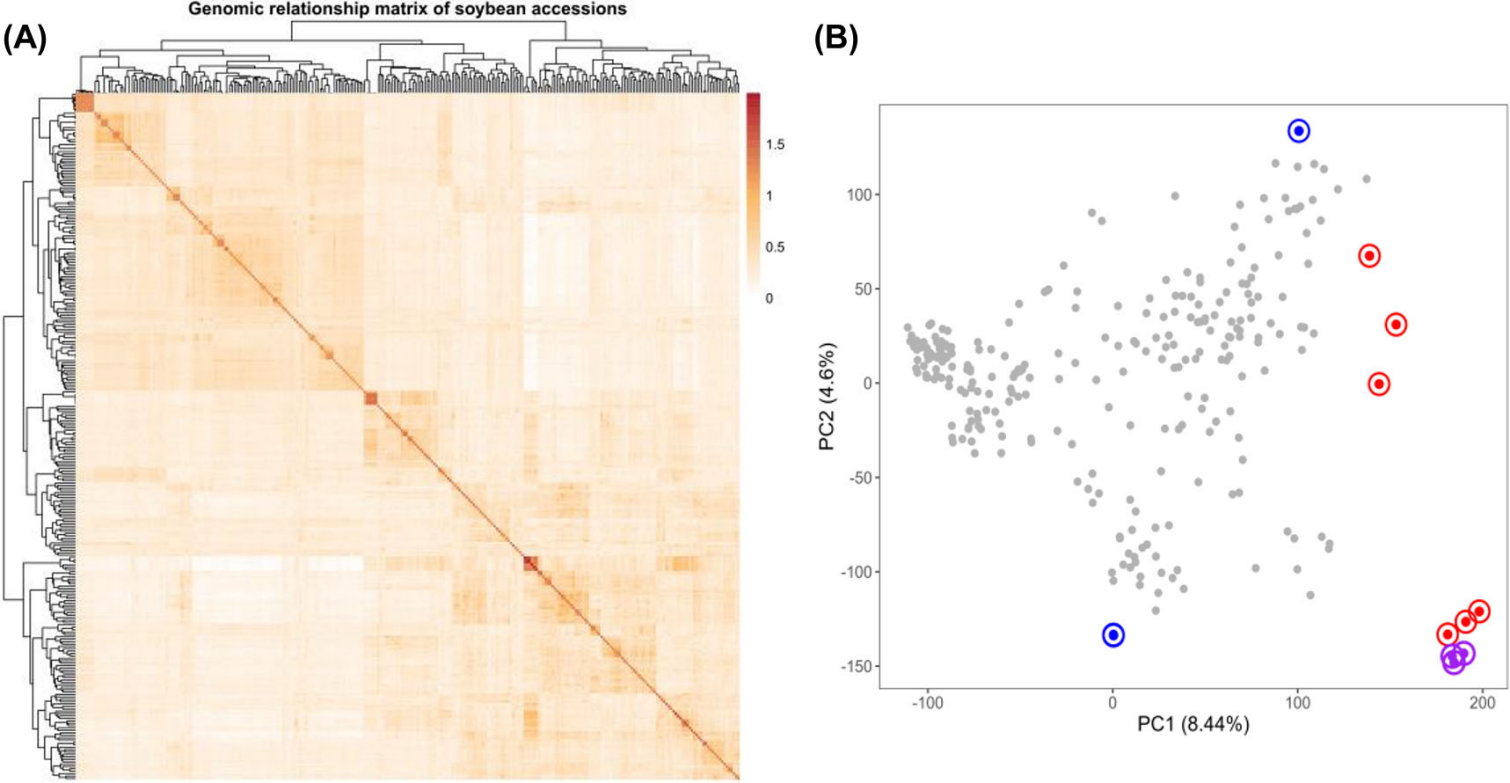
**(A)** Genomic relationship matrix of soybean accessions inferred from genome-wide SNP genotypes. Pairwise genomic relationship coefficients were calculated using genotype data and visualized as a heatmap, with darker colors indicating higher genetic similarity. Hierarchical clustering was applied to both rows and columns based on the genomic relationship matrix. **(B)** Principal component analysis (PCA) of soybean accessions based on genome-wide SNP genotypes prior to GWAS. The first two principal components (PC1 and PC2) are shown, explaining 8.44% and 4.60% of the total genetic variation, respectively. Accessions with extreme values along PC1 and PC2 (i.e., those located at the outer edges of the distribution) are highlighted for clarity. Blue circles indicate accessions with extreme PC1 values, red circles indicate accessions with extreme PC2 values, and purple circles indicate accessions located in the lower-right region of the plot (high PC1 and low PC2).

A genomic relationship matrix (GRM) was generated using a set of 68,375 genome-wide SNP markers to assess genetic relatedness among 286 soybean accessions. The kinship heatmap showed a clear diagonal pattern reflecting high self-relatedness and indicating that the marker set provided consistent estimates of genomic relationships (**Figure 2A**). Off-diagonal kinship values were generally low to moderate, suggesting that most accessions were not closely related. No obvious separation of the panel into highly differentiated genetic clusters was evident from the kinship patterns alone. The PCA revealed that PC1 and PC2 explained 8.51% and 4.52% of the total genetic variation, respectively, followed by a gradual decline in variance explained by subsequent components (**Figure 2B**). The PC1–PC2 scatter plot did not reveal any clear discrete subpopulation structure, indicating that the panel retains broad genetic variation while maintaining only moderate background relatedness among accessions.

### Genome-wide association study for canopy wilting response

**Figure 3:**
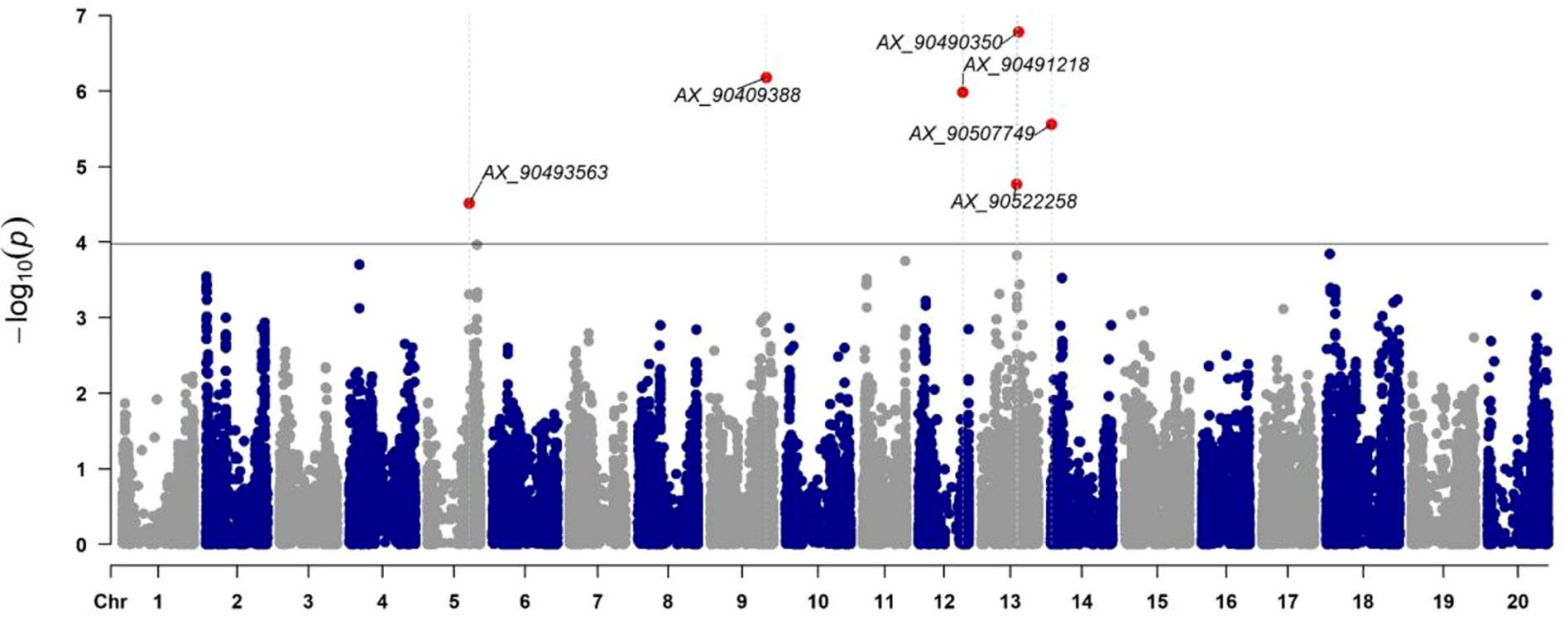
Manhattan plot of genome-wide association results for canopy wilting in 286 cultivated soybean accessions. Each point represents a single-nucleotide polymorphism (SNP) plotted according to its genomic position across 20 chromosomes (*x*-axis) and the −log_10_(*p*) value obtained from the BLINK model (*y*-axis). Blue and gray points indicate SNPs on alternating chromosomes. Red points represent SNPs exceeding the predefined significance threshold (−log_10_(*p*) ≥ 4) and are labeled with their marker IDs.

A total of six significant SNPs associated with canopy wilting response were identified on chromosomes (Chrs.) 5, 9, 12, 13, and 14 (**Figure 3**; **Table 3**). PVE (%) represents the proportion of phenotypic variance explained by each SNP, indicating the relative contribution of each locus to canopy wilting. The PVE values of the identified SNPs ranged from 0.59% to 12.69%, indicating that these loci have varying contributions to canopy wilting, with several SNPs showing moderate effects. Among them, AX-90490350 located at 29,572,960 bp on Chr. 13 was the most significant SNP [−log_10_(*p*) = 6.8], followed by AX-90409388 located at 44,139,963 bp on Chr. 9 [−log_10_(*p*) = 6.2], while AX-90493563 mapped at 33,165,189 bp on Chr. 5 showed the lowest −log_10_(*p*) value (4.5). The minor allele frequency of the significant SNPs ranged from 0.2 (AX-90493563) to 0.4 (AX-90491218).

**Table 3:** Significant SNPs associated with canopy wilting identified by BLINK genome-wide association analysis using adjusted mean values.

Significant SNP	Chr	Position (bp)	Favorable allele	Unfavorable allele	−log_10_(*p*)	MAF	PVE (%)	Adjusted mean (favorable)	Adjusted mean (unfavorable)	Favorable (*n*)	Unfavorable (*n*)
AX-90493563	5	33,165,189	GG	AA	4.52	0.196	0.59	3.19	3.3	230	56
AX-90409388	9	44,139,963	TT	CC	6.18	0.248	9.50	2.92	3.31	71	215
AX-90491218	12	34,911,811	GG	TT	5.99	0.420	3.11	3.10	3.30	119	164
AX-90522258	13	27,987,504	TT	GG	4.77	0.388	12.69	3.06	3.46	175	111
AX-90490350	13	29,572,960	TT	GG	6.79	0.311	10.71	3.09	3.48	197	89
AX-90507749	14	321,593	GG	CC	5.56	0.315	6.01	3.02	3.30	90	196

For each SNP, the chromosome (Chr), physical position (bp), favorable and unfavorable alleles, −log10(*p*) value, minor allele frequency (MAF), proportion of phenotypic variance explained (PVE), adjusted mean values for each allelic class (favorable and unfavorable), and the number of accessions carrying each allele (*n*) are presented. Favorable alleles were defined as those associated with lower canopy wilting scores based on adjusted mean values.

### Allelic effect of significant SNPs

**Figure 4:**
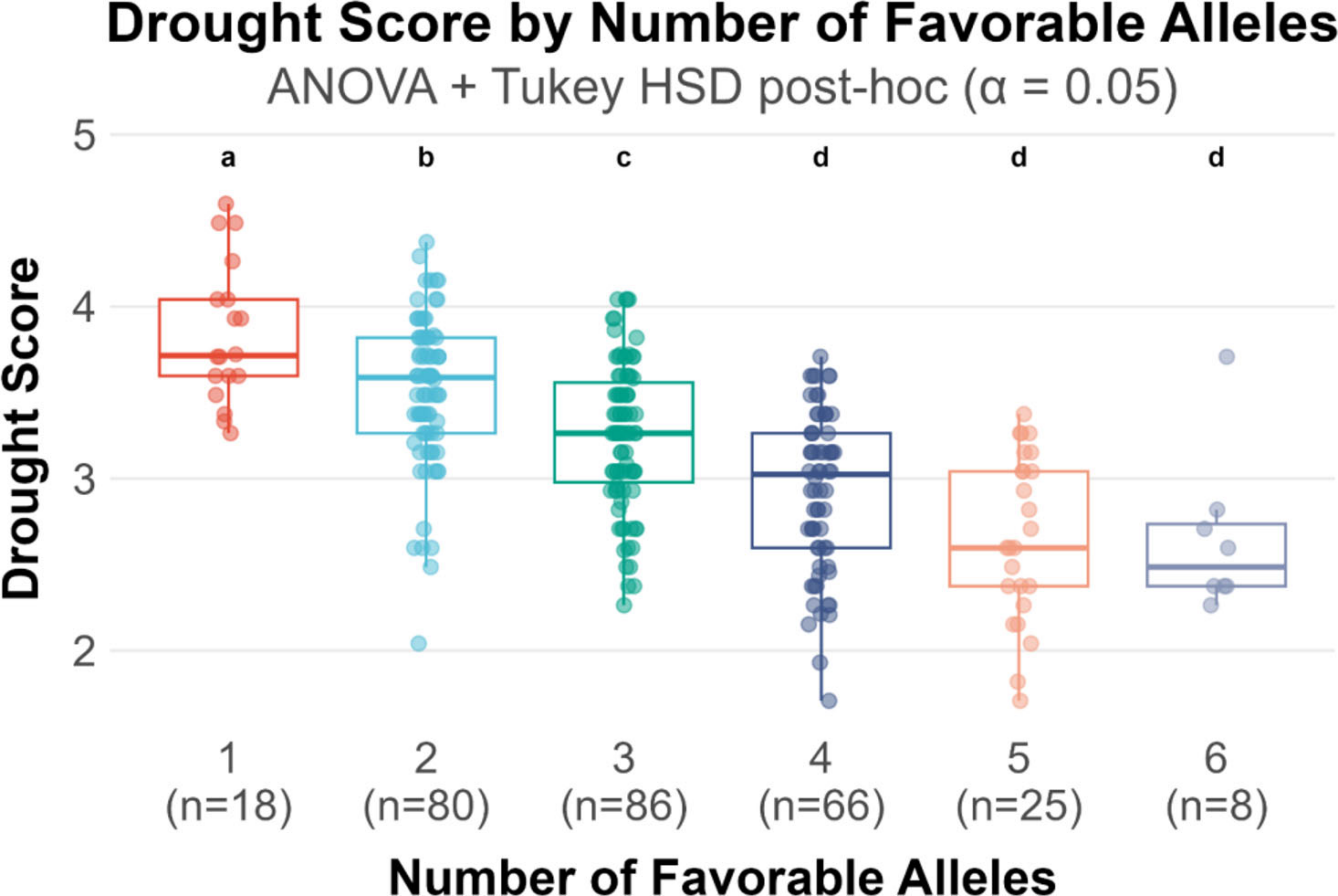
**A** canopy wilting score variation based on the number of favorable alleles within the diverse panel. A different alphabet indicates a significant difference in the canopy wilting score between the two groups, based on the Tukey HSD *post hoc* test (α = 0.05).

Favorable alleles were defined as those associated with lower canopy wilting scores (i.e., reduced wilting under drought stress), whereas unfavorable alleles were associated with higher wilting scores. The favorable and unfavorable alleles for each significant SNP were determined based on the average of adjusted means across the diverse panel. The favorable and unfavorable alleles for each significant SNP were defined using the average of adjusted means among the diverse panel. The phenotypic variations were evaluated based on the number of favorable alleles (**Figure 4**). Accessions with heterozygosity at the significant SNP position were excluded from this analysis. Notably, the accessions with more favorable alleles showed greater tolerance to drought stress based on canopy wilting response. The accessions with only one favorable allele showed high canopy wilting scores on average (3.8), indicating high susceptibility to drought stress. On the other hand, the accessions with six favorable alleles showed a low canopy wilting score on average (2.6), indicating tolerance to stress.

### Identification of potential candidate genes

A total of 36 candidate genes were identified within LD blocks containing significant SNPs on Chrs. 5, 9, 12, 13, and 14 (**Table 4**). Because of no LD block of AX-90490350, a candidate gene that was adjacent was selected, which was *Glyma.13G182600*. Overall, the size of LD blocks ranged from 23.9 kbp (Chr. 9) to 99.9 kbp (Chr. 12). The LD block harboring the largest number of candidate genes (14 genes) was on Chr. 14, whereas the one with the lowest number of candidate genes (three genes) was on Chr. 5. Among 36 candidate genes, leucine-rich repeat receptor-like protein kinase (*Glyma.05G139100*, *Glyma.12G188100*, and *Glyma.14G002400*) and PPR repeat-containing protein (*Glyma.12G187900*, *Glyma.12G188000*, and *Glyma.14G003000*) were the most frequently identified functional annotations, followed by aluminum-activated malate transporter (*Glyma.12G188400* and *Glyma.12G188500*). Six candidate genes were annotated as proteins of unknown function or lacked conserved domain annotations. The remaining candidate genes represented diverse functional categories, including receptor-like protein kinases, protein phosphatases, transcription-related proteins, RNA processing factors, and vesicle trafficking-associated proteins.

**Table 4:** Putative candidate genes identified within LD-defined genomic regions surrounding significant SNPs associated with canopy wilting.

Significant SNP	Gene ID	Location (bp)	Predicted function/domain
AX-90493563	Glyma.05g139100	33,165,320–33,165,996	Leucine-rich repeat receptor-like protein kinase (LRR-RLK)
	Glyma.05g139200	33,173,197–33,181,398	Protein of unknown function
	Glyma.05g139300	33,182,337–33,186,879	Enolase superfamily/mandelate racemase-like protein
AX-90409388	Glyma.09g218100	44,122,773–44,131,298	GMC oxidoreductase family protein
	Glyma.09g218300	44,132,685–44,135,293	Protein of unknown function
	Glyma.09g218400	44,137,379–44,143,985	SCD6-related protein
	Glyma.09g218500	44,149,101–44,157,808	Adaptin family protein (AP complex subunit)
AX-90491218	Glyma.12g187700	34,879,228–34,887,037	NIMA-related serine/threonine protein kinase (NEK)
	Glyma.12g187900	34,904,635–34,905,721	PPR repeat-containing protein
	Glyma.12g188000	34,905,867–34,907,438	PPR repeat-containing protein
	Glyma.12g188100	34,912,304–34,916,479	Leucine-rich repeat receptor-like protein kinase (LRR-RLK)
	Glyma.12g188200	34,921,704–34,925,036	Histone deacetylase (HDAC)
	Glyma.12g188300	34,938,827–34,948,249	Mediator complex subunit 8 (MED8)
	Glyma.12g188400	34,956,686–34,959,969	Aluminum-activated malate transporter (ALMT)
	Glyma.12g188500	34,986,837–34,988,651	Aluminum-activated malate transporter (ALMT)
	Glyma.12g188600	34,993,429–35,001,448	WNK (With No Lysine)-related serine/threonine protein kinase-like protein
AX-90522258	Glyma.13g164900	27,975,423–27,977,287	Protein of unknown function
	Glyma.13g165000	27,987,286–27,988,809	Protein with no annotated conserved domain
	Glyma.13g165100	27,991,996–27,994,467	Ionotropic glutamate receptor-like protein
	Glyma.13g165200	27,997,373–27,999,343	40S ribosomal protein S11
	Glyma.13g165300	28,011,615–28,015,224	Protein phosphatase 2C
AX-90490350	Glyma.13G182600	29,565,815–29,573,222	Spliceosome-associated protein
AX-90507749	Glyma.14g002200	251,616–252,376	No conserved domain annotated
	Glyma.14g002300	253,744–258,686	DUF630
	Glyma.14g002400	262,666–269,931	Leucine-rich repeat receptor-like protein kinase (LRR-RLK)
	Glyma.14g002500	276,726–279,703	UDP-glucosyl/glucuronosyl transferase
	Glyma.14g002600	280,278–282,577	No conserved domain annotated
	Glyma.14g002700	284,497–291,587	RING finger protein (C3HC4)
	Glyma.14g002800	293,552–294,736	VQ motif-containing protein
	Glyma.14g002900	296,230–299,990	Choline-phosphate cytidylyltransferase
	Glyma.14g003000	300,925–303,345	PPR repeat-containing protein
	Glyma.14g003100	304,142–306,158	AAA+ ATPase
	Glyma.14g003200	306,245–310,111	Coproporphyrinogen III oxidase
	Glyma.14g003300	312,114–315,407	GNAT-family N-acetyltransferase
	Glyma.14g003400	317,498–319,208	Chlorophyll a/b binding protein
	Glyma.14g003500	320,046–323,715	Endomembrane protein 70

Genes located within LD blocks containing significant SNPs were selected based on their physical positions in the Glycine max reference genome (Wm82.a2.v1). Functional annotations and predicted protein domains were obtained from SoyBase.

### Tissue-specific expression patterns of candidate genes

**Figure 5:**
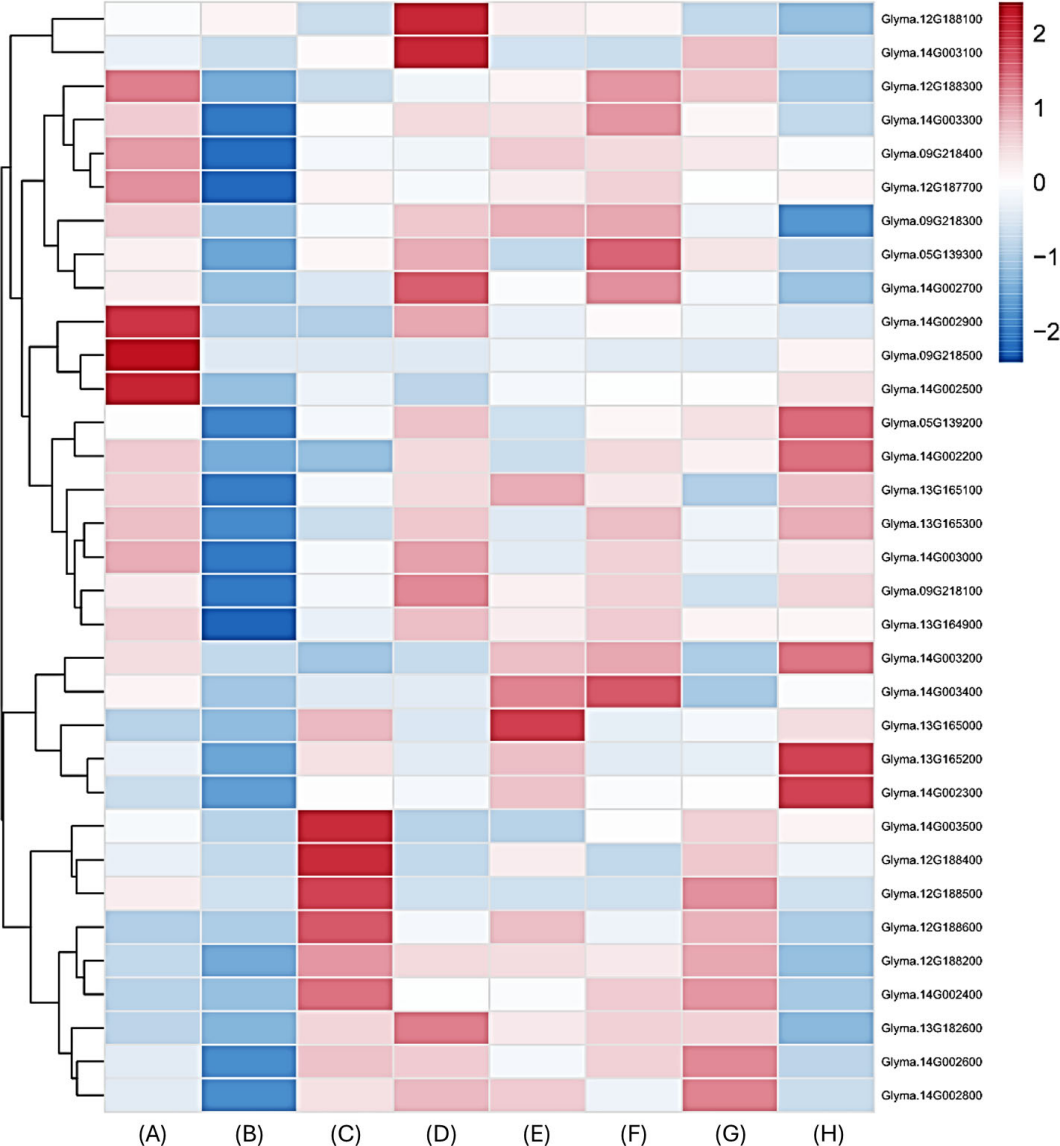
Heatmap of tissue-specific expression patterns of candidate genes in soybean. Gene expression levels across different tissues—root **(A)**, radicle **(B)**, leaf **(C)**, petiole **(D)**, seedling **(E)**, hypocotyl **(F)**, shoot **(G)**, and nodule **(H)**—were obtained from soybean expression datasets and summarized as mean transcripts per million (TPM) values. Mean TPM values were log_2_-transformed [log_2_(TPM + 1)], scaled by gene, and visualized as a heatmap, with red and blue indicating higher and lower expression levels, respectively.

The tissue-specific RNA-seq atlas analysis revealed gene expression patterns of candidate genes associated with canopy wilting response in soybean (**Figure 5**). Notably, three genes, *Glyma.14G002900*, *Glyma.09G218500*, and *Glyma.14G002500*, showed relatively higher gene expression levels in root tissue, while two genes, *Glyma.13G165200* and *Glyma.14G002300*, showed relatively higher gene expression levels in nodule, whereas two genes, *Glyma.14G003500* and *Glyma.12G188400*, were highly expressed in leaf and moderately expressed in shoot tissues. Two genes, *Glyma.12G188100* and *Glyma.14G003100*, showed higher expression levels in petiole tissue. All candidate genes showed very low gene expression levels in radicle tissue.

## Discussion

### Year and developmental effects on canopy wilting

Over three consecutive years (2021–2023), 286 diverse soybean accessions were evaluated for canopy wilting under drought stress in a rainout-shelter greenhouse. Although phenotyping dates varied slightly among years, soybean developmental stage is primarily determined by thermal time accumulation and photoperiod sensitivity rather than calendar date. Approximately 75% of the accessions belonged to MG IV and V and were predominantly at early reproductive stages at the time of evaluation based on maturity classification and planting schedules. Furthermore, the weak association between days to maturity and canopy wilting score (*R*² = 0.07) suggests that the influence of maturity on canopy wilting was likely limited under the conditions of this study.

Although year-to-year correlations of genotype means were low (*r* = −0.02 to 0.18), GWAS was performed using adjusted mean values across years, which account for environmental heterogeneity and better isolate genotypic effects. The relatively low correlations among years further suggest that canopy wilting reflects dynamic physiological responses to environmental conditions rather than fixed developmental differences. Collectively, these observations support the interpretation of canopy wilting as a complex quantitative trait influenced by multiple physiological processes related to plant water regulation under drought conditions (Charlson et al., 2009; Hwang et al., 2016).

### Environmental influence on canopy wilting

Substantial phenotypic variation in canopy wilting scores was observed across years, reflecting the sensitivity of this trait to environmental conditions. In rainout-shelter greenhouse settings, natural fluctuations in air humidity and vapor pressure deficit (VPD) are difficult to completely standardize and may have contributed to differences in stress intensity among years. Previous studies have demonstrated that variation in air humidity can significantly influence soybean growth and physiological performance, even under irrigated conditions, affecting traits such as biomass accumulation, pod number, and photosynthetic activity (Woodward and Begg, 1976). Moreover, the influence of VPD on transpiration and photosynthesis involves not only stomatal regulation but also genotype-specific physiological adjustments (Zou and Kahnt, 1988). Such environmental responsiveness is consistent with the complex physiological basis of canopy wilting, which integrates dynamic water-use processes under drought stress. Despite these sources of environmental variation, several accessions consistently exhibited favorable wilting responses across years, indicating stable genotypic differences (**Supplementary Table 4**). These accessions therefore represent valuable germplasm resources that could be utilized as parental materials in breeding programs aimed at enhancing drought tolerance through improved canopy water regulation.

### Genome-wide association study

The reliability of the GWAS results was assessed using a quantile–quantile (QQ) plot and the genomic inflation factor (λ). The QQ plot showed that the observed *p*-values generally followed the expected distribution under the null hypothesis, with noticeable deviations only in the tail region, suggesting the presence of true marker–trait associations. The genomic inflation factor (λ) was estimated to be 1.08, indicating minimal inflation of test statistics and effective control of false positives. Together, these results suggest that population structure and relatedness were adequately controlled, supporting the robustness of the BLINK model used in this study.

Based on the validated GWAS result, all significant SNPs identified in this study were mapped within ±1 Mbp of genomic regions previously reported to be associated with canopy wilting (**Table 3**). The SNP AX-90493563 on Chr. 5 (33.17 Mbp) was located near ss715590864 (33.18 Mbp) reported by Kaler et al. (2017), supporting the reproducibility of this genomic region across independent populations. Similarly, AX-90409388 on Chr. 9 (44.14 Mbp) was located near previously reported SNPs ss715604746 (43.75 Mbp; Kaler et al., 2017) and ss715604845 (44.80 Mbp; Chamarthi et al., 2021), suggesting a stable genomic region for canopy wilting. A significant SNP on Chr. 12, AX-90491218 (34.91 Mbp), was located approximately 800 kbp downstream from the previously reported SNP ss715612366 (34.10 Mbp) (Chamarthi et al., 2021). Likewise, two significant SNPs on Chr. 13, AX-90522258 (27.99 Mbp) and AX-90490350 (29.57 Mbp), were located near previously reported SNPs ss715614911 (28.88 Mbp; Chamarthi et al., 2021) and ss715614803 (29.46 Mbp; Steketee et al., 2020), indicating that Chr. 13 harbors multiple conserved regions associated with canopy wilting. The SNP AX-90507749 on Chr. 14 (0.32 Mbp) was also identified within 1 Mbp of ss715620072 (0.92 Mbp) reported by Chamarthi et al. (2021). Collectively, the consistent physical proximity between the significant SNPs identified in this study and previously reported loci provides independent support for the relevance of these genomic regions in controlling canopy wilting in soybean. The significant SNPs identified in this study primarily provide independent validation of previously reported genomic regions associated with canopy wilting. The close physical proximity between these SNPs and previously reported markers suggests a refinement of these loci and supports their stability across different populations and environmental conditions.

### Identification of candidate genes

Within the LD blocks of significant SNPs, a total of 36 candidate genes were located within the LD blocks for each significant SNP on Chrs. 5, 9, 12, 13, and 14. Among them, three genomic regions containing leucine-rich repeat receptor-like protein kinase (*Glyma.05G139100*, *Glyma.12G188100*, and *Glyma.14G002400*) were identified on Chrs. 5, 12, and 14. The leucine-rich repeat receptor-like protein kinase is an important membrane-localized receptor that is responsible for plant regulatory responses by interlinking abscisic acid and brassinosteroid signaling, stomatal movement, and reactive oxygen species (ROS) homeostasis under water-limited conditions (Yan et al., 2025). In particular, two genes, *Glyma.12G188100* and *Glyma.14G002400*, also showed higher gene expression levels in the petiole and leaf, respectively, suggesting that these candidate genes may play important roles in regulating canopy wilting in soybean. Furthermore, three PPR repeat-containing protein-encoding genes (*Glyma.12G187900*, *Glyma.12G188000*, and *Glyma.14G003000*) were also identified on Chrs. 12 and 14. A PPR repeat-containing protein plays an important role in RNA editing, plant growth and development, and responses to abiotic stresses (Xing et al., 2018; Su et al., 2019). Another gene, *Glyma.12g188600*, encoding a WNK (With No Lysine)-related serine/threonine protein kinase-like protein, was identified within a significant genomic region on Chr. 12 and showed relatively higher gene expression level in leaf tissues. This gene has previously been reported as a candidate gene associated with leaf pubescence length and density in soybean (Li et al., 2022). Leaf pubescence influences plant water relations by modifying the leaf boundary layer and reducing transpirational water loss. Consistent with this, pubescence density has been reported to play an important role in soybean drought tolerance and plant water status (Du et al., 2009). Therefore, the association of *Glyma.12g188600* with pubescence-related traits, together with its proximity to a canopy wilting-associated locus identified in this study, suggests that this gene may contribute to drought responses through leaf surface-mediated regulation of transpiration rather than through direct stress signaling pathways. A candidate gene identified on Chr. 14, *Glyma.14g003200*, encodes coproporphyrinogen III oxidase, a key enzyme in heme biosynthesis, and showed preferential expression in nodules, where metabolic activity and redox regulation are tightly linked to nitrogen fixation and stress sensitivity. Variation in heme-associated metabolic processes has been suggested to influence redox balance and cellular energy status, which may affect plant performance under water-limited conditions. This gene also exhibited a tissue-specific expression pattern in nodule.

### Functional relevance of candidate genes based on tissue-specific expression

The tissue-specific expression patterns of candidate genes provide insights into potential mechanisms underlying canopy wilting in soybean. Root-expressed genes, including Glyma.09g218500 and Glyma.14g002500, may be associated with water uptake and hormone-related responses under drought conditions. Genes preferentially expressed in petiole tissues, such as Glyma.12g188100 and Glyma.14g003100, may be involved in signal transduction and transport processes between plant organs. Leaf-expressed genes, including Glyma.12g188400 and Glyma.14g003500, may contribute to transpiration-related processes, potentially affecting water loss and canopy wilting. In particular, Glyma.12g188100 (LRR-RLK), which is highly expressed in petiole tissue, may be involved in sensing drought-related signals and transmitting them between roots and leaves. Glyma.09g218500 (adaptin family protein), expressed in root tissue, may regulate vesicle trafficking and membrane protein localization, potentially influencing water uptake processes. Glyma.12g188400 (ALMT), highly expressed in leaf tissue, may be associated with stomatal regulation and ion transport, thereby affecting transpiration and water loss. Genes expressed in nodules, such as Glyma.13g165200 and Glyma.14g002300, may be related to general metabolic activity under stress conditions. These results suggest that canopy wilting may be influenced by multiple processes, including water uptake, transport, and loss, with tissue-specific expression patterns providing supportive evidence for their potential roles.

### Limitations

This study has several limitations that should be considered. A well-watered control was not included, which limits the ability to distinguish drought-specific responses from inherent genotype differences. However, the use of controlled drought conditions allows for the effective evaluation of genotypic variation in canopy wilting. In addition, variation in maturity among accessions may have influenced canopy wilting to some extent, although the relatively weak relationship observed between these traits suggests a limited effect. Environmental variation across years was not fully characterized, which may affect genotype × environment interpretation. Moreover, the number of significant SNPs and their effect sizes were relatively limited, suggesting that canopy wilting is controlled by a complex genetic architecture. The overlap with previously reported regions indicates that this study primarily provides validation rather than the discovery of novel loci. In addition, canopy wilting represents only one aspect of drought tolerance, and the inclusion of additional physiological or yield-related traits would provide a more comprehensive understanding of drought response. Finally, results obtained under greenhouse conditions may not fully translate to field environments.

### Future direction

Further studies are expected to provide the validation and refinement needed for the genomic regions identified in this study. As a next step, targeted resequencing of selected accessions exhibiting strong drought tolerance or susceptibility would enable detailed characterization of SNP and INDEL variation within these regions, helping to resolve allelic differences associated with canopy wilting responses. In addition, analysis of copy number variation (CNV) across the identified loci may provide further insight into potential gene dosage effects contributing to drought-related phenotypic variation. Transcriptome-based approaches, including RNA sequencing of representative tolerant and susceptible accessions, would also be useful for assessing expression patterns of the proposed candidate genes under drought stress. Ultimately, integrating sequence variation, gene dosage, and expression data will be critical for establishing causal links between candidate genes and canopy wilting responses, and for advancing the functional validation of key loci relevant to soybean drought tolerance.

## Conclusion

The GWAS analysis using 286 diverse accessions identified six significant SNPs on Chrs. 5, 9, 12, 13, and 14, which are associated with drought tolerance regarding the canopy wilting response in soybean. Collectively, the identified candidate genes include receptor-like kinases, RNA-processing-related proteins, and stress-responsive metabolic enzymes, suggesting that multiple signaling and regulatory pathways contribute to canopy wilting responses in soybean. The novel genetic resources for drought tolerance identified in this study will benefit soybean breeders and growers by supporting sustained yield production and facilitating the development of new cultivars.

## Data Availability

The original contributions presented in the study are included in the article/**Supplementary Material**. Further inquiries can be directed to the corresponding author.
