# Supplementary figures and images for: Genome-wide association study on soybean canopy wilting under drought stress conditions in a rainout-shelter greenhouse
Source: Front Plant Sci. 2026 Jun 9;17:1840313. doi: 10.3389/fpls.2026.1840313 (PMC13287008; doi:10.3389/fpls.2026.1840313)

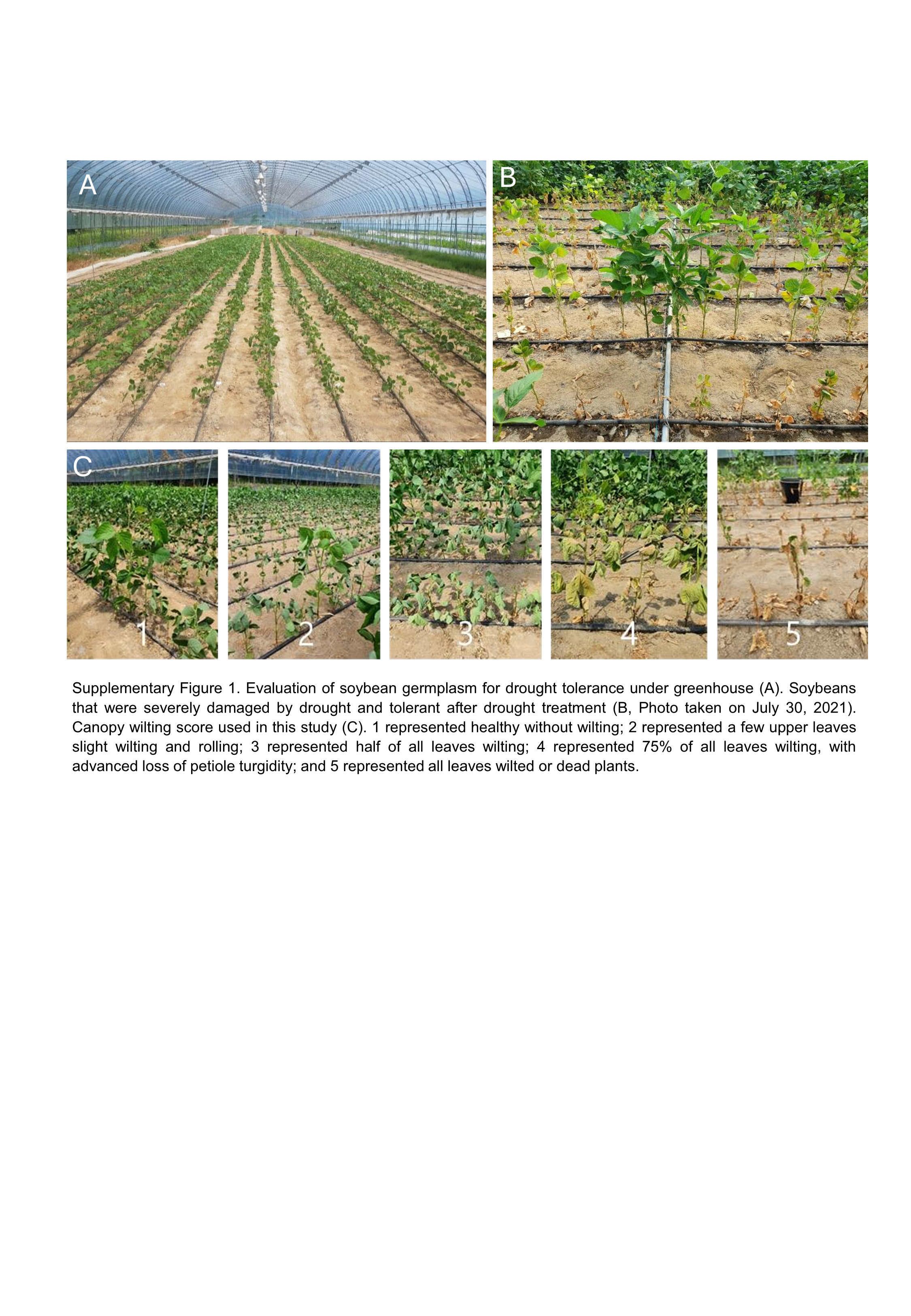

Supplement: Supplementary file 1 [file Image1.tif]

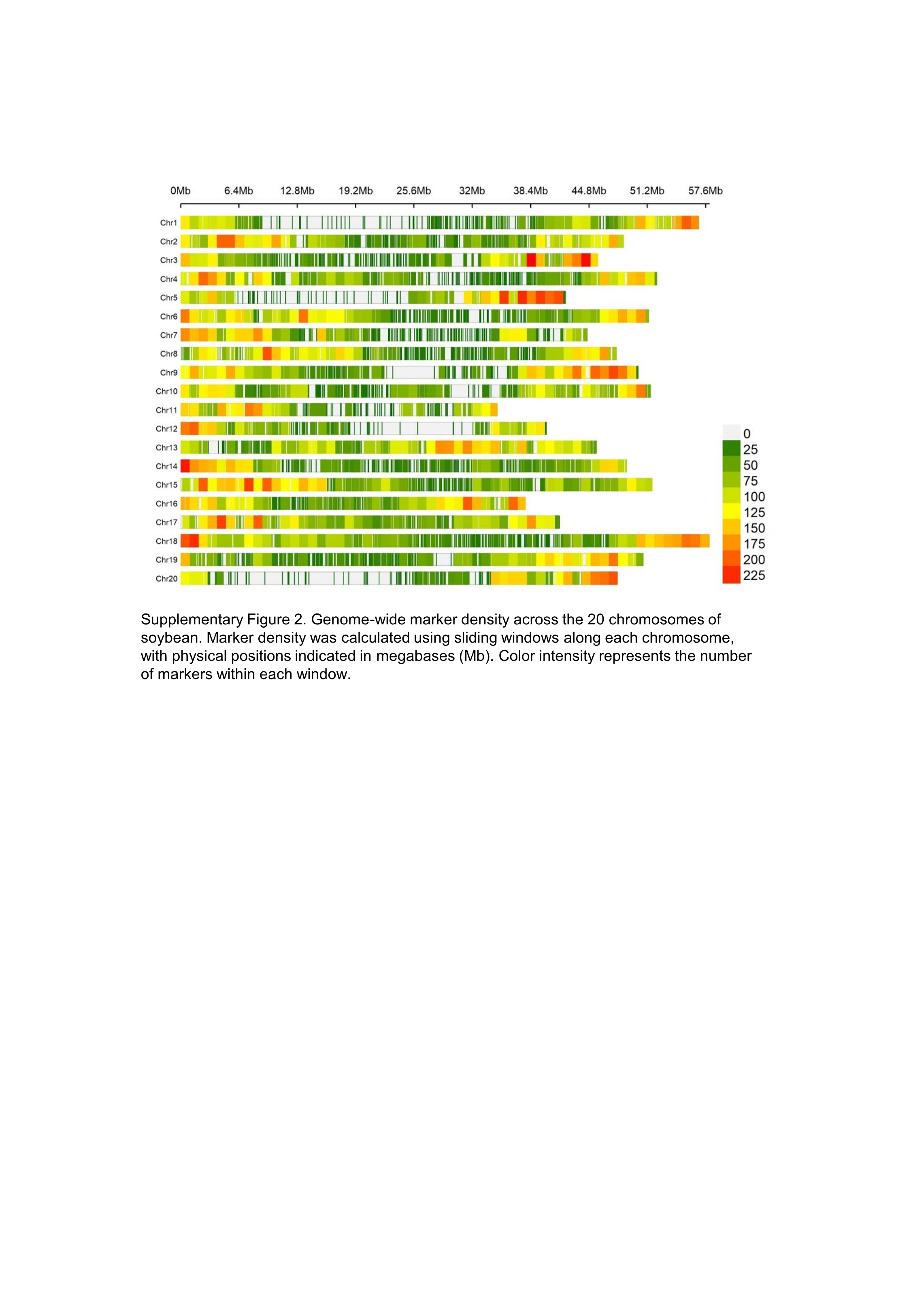

Supplement: Supplementary file 2 [file Image2.tif]

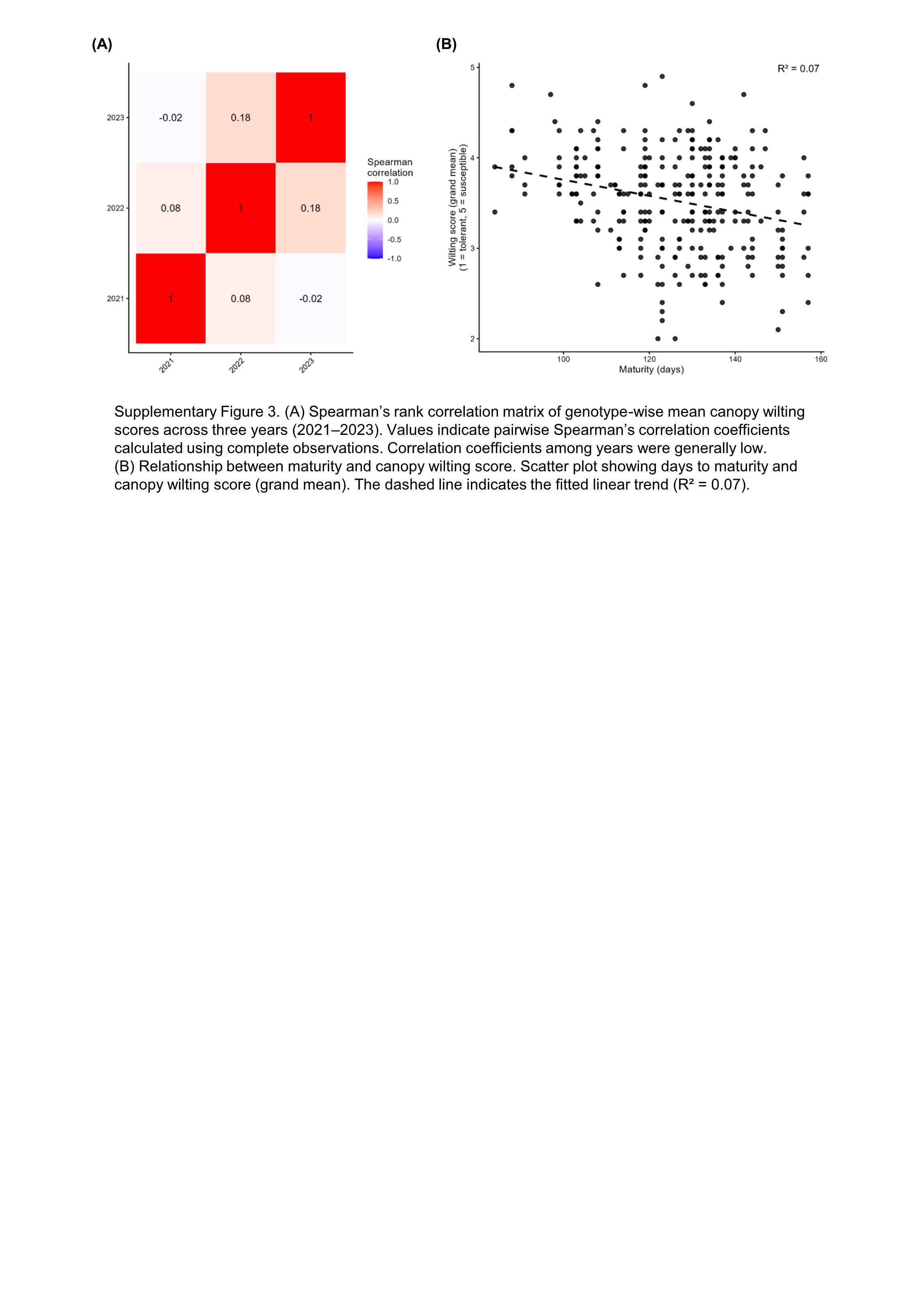

Supplement: Supplementary file 3 [file Image3.tif]

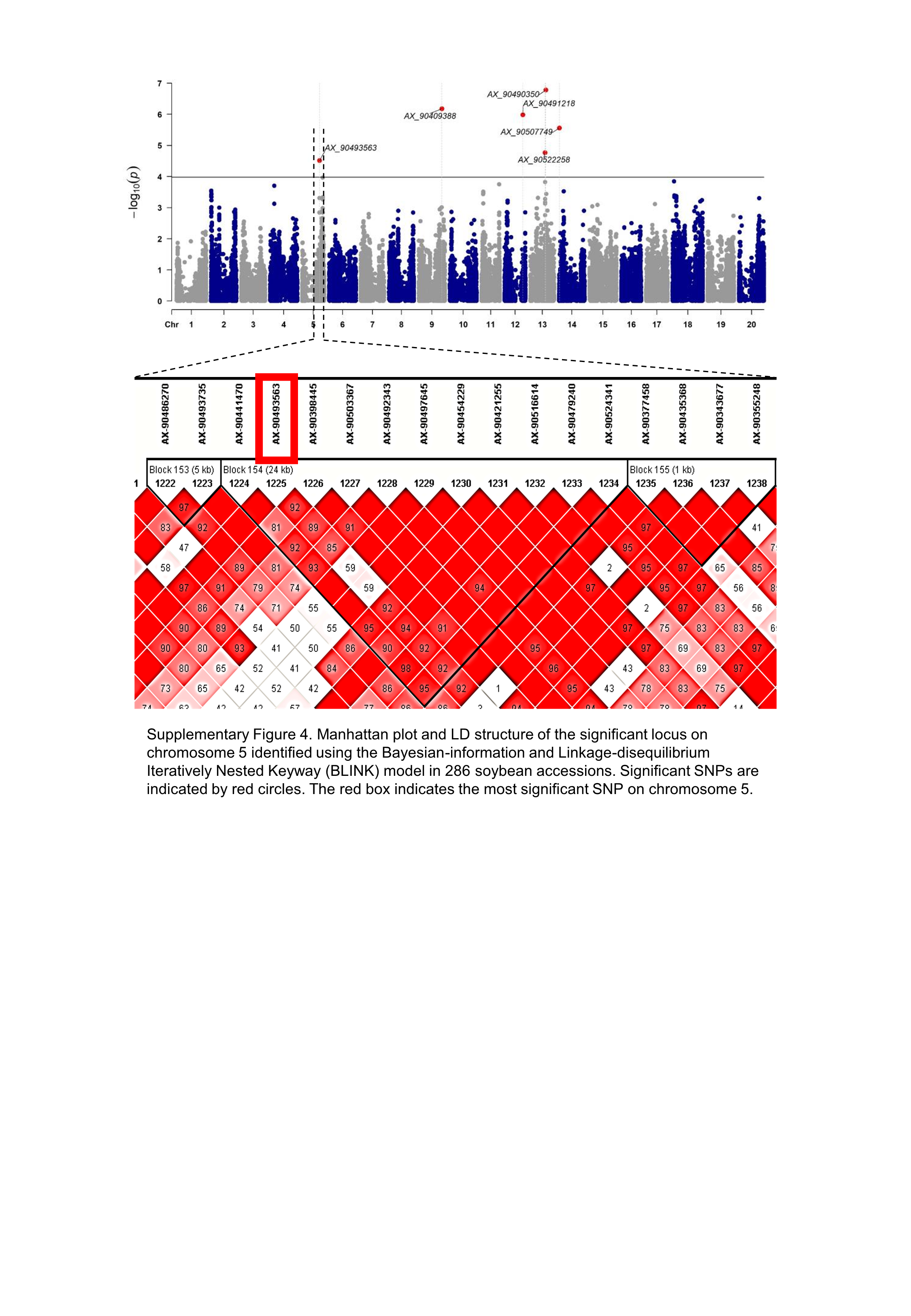

Supplement: Supplementary file 4 [file Image4.tif]

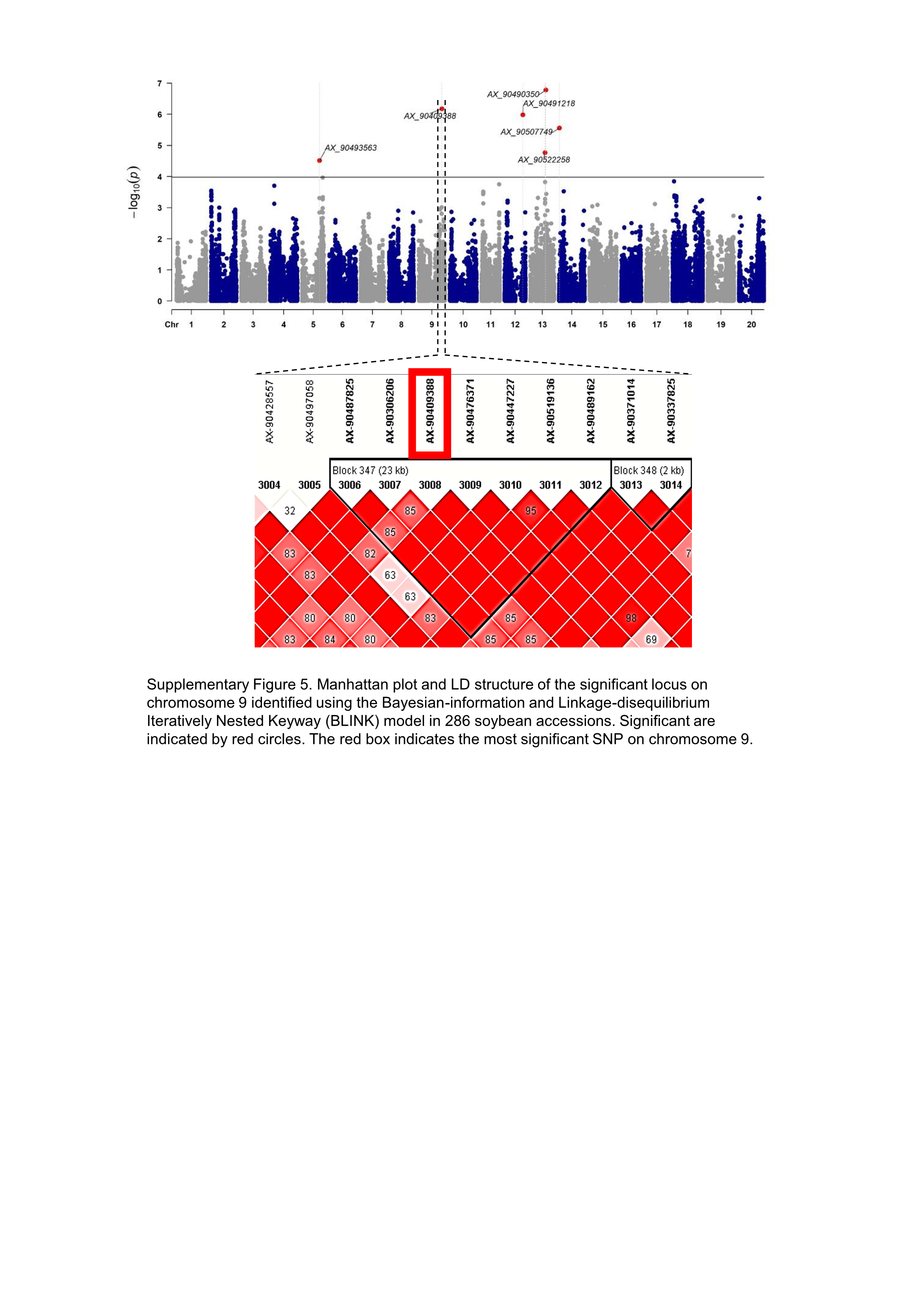

Supplement: Supplementary file 5 [file Image5.tif]

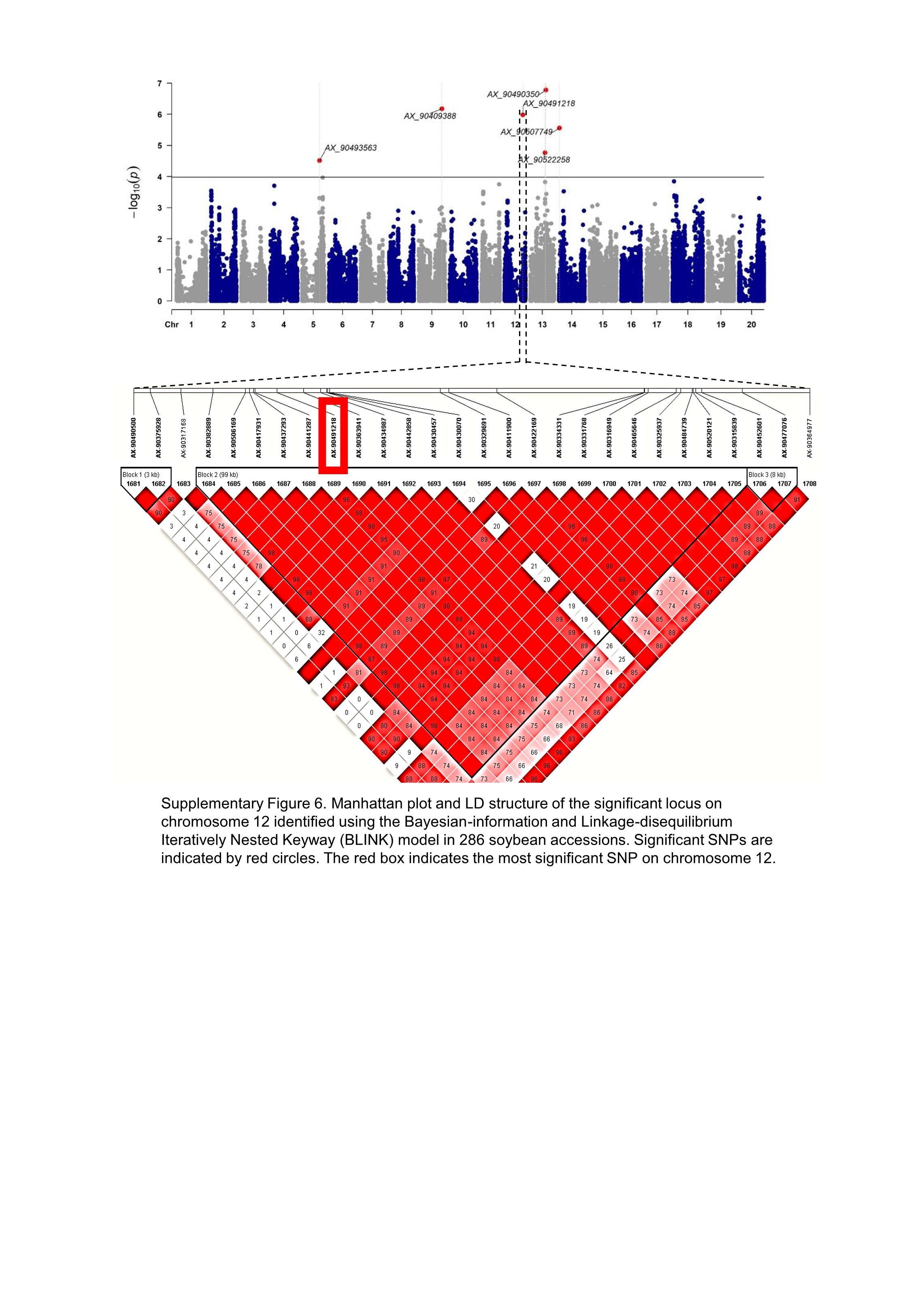

Supplement: Supplementary file 6 [file Image6.tif]

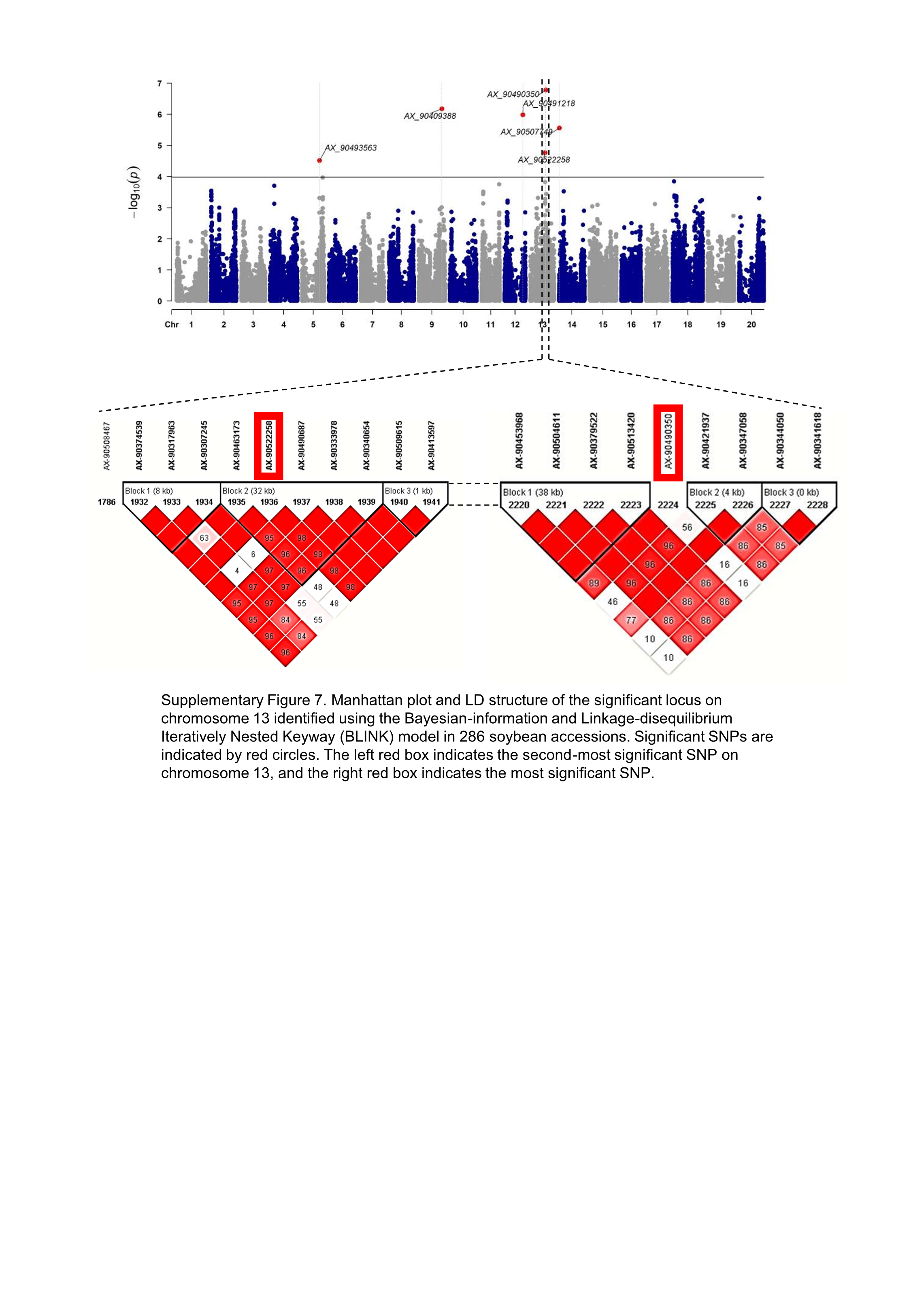

Supplement: Supplementary file 7 [file Image7.tif]

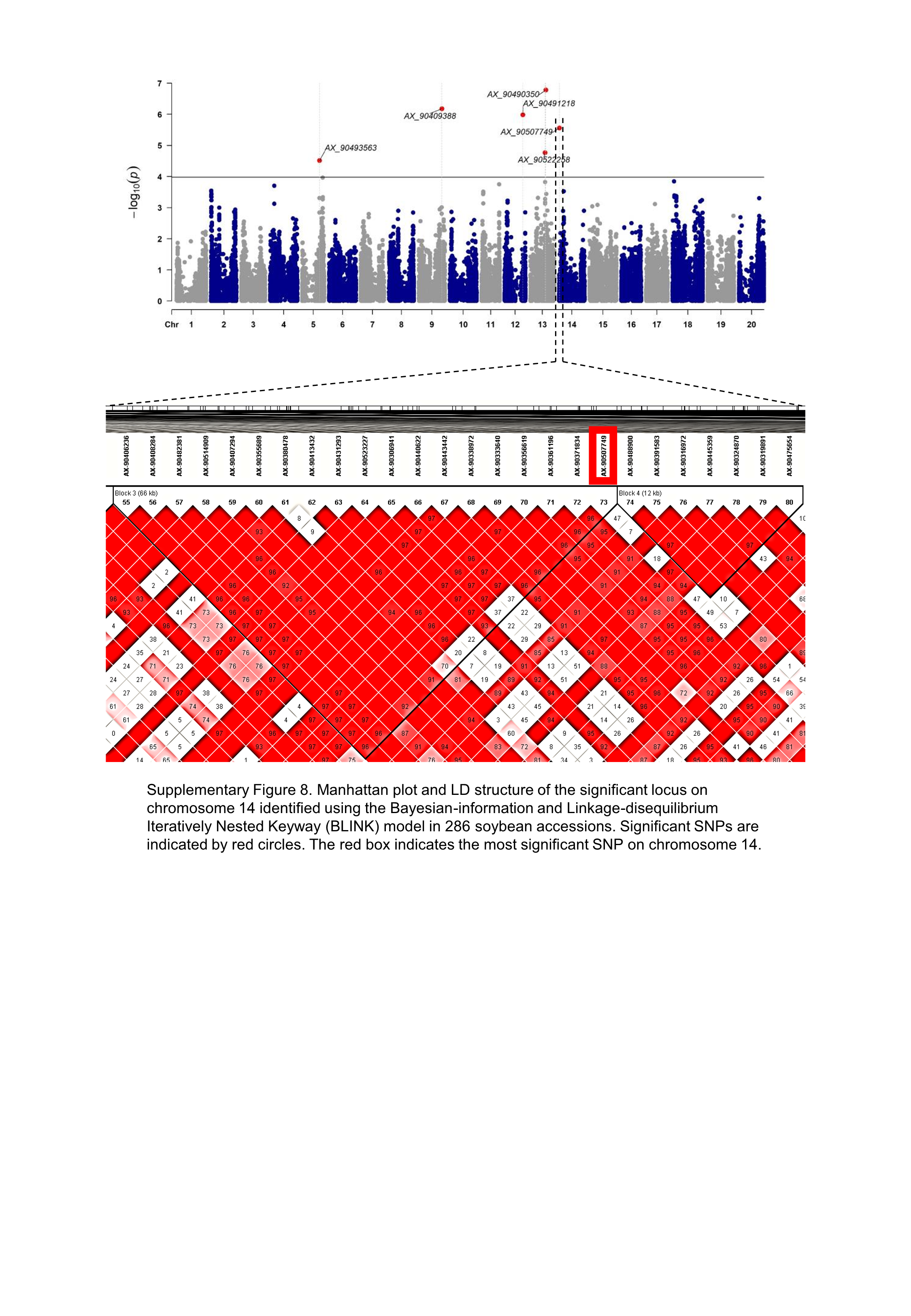

Supplement: Supplementary file 8 [file Image8.tif]

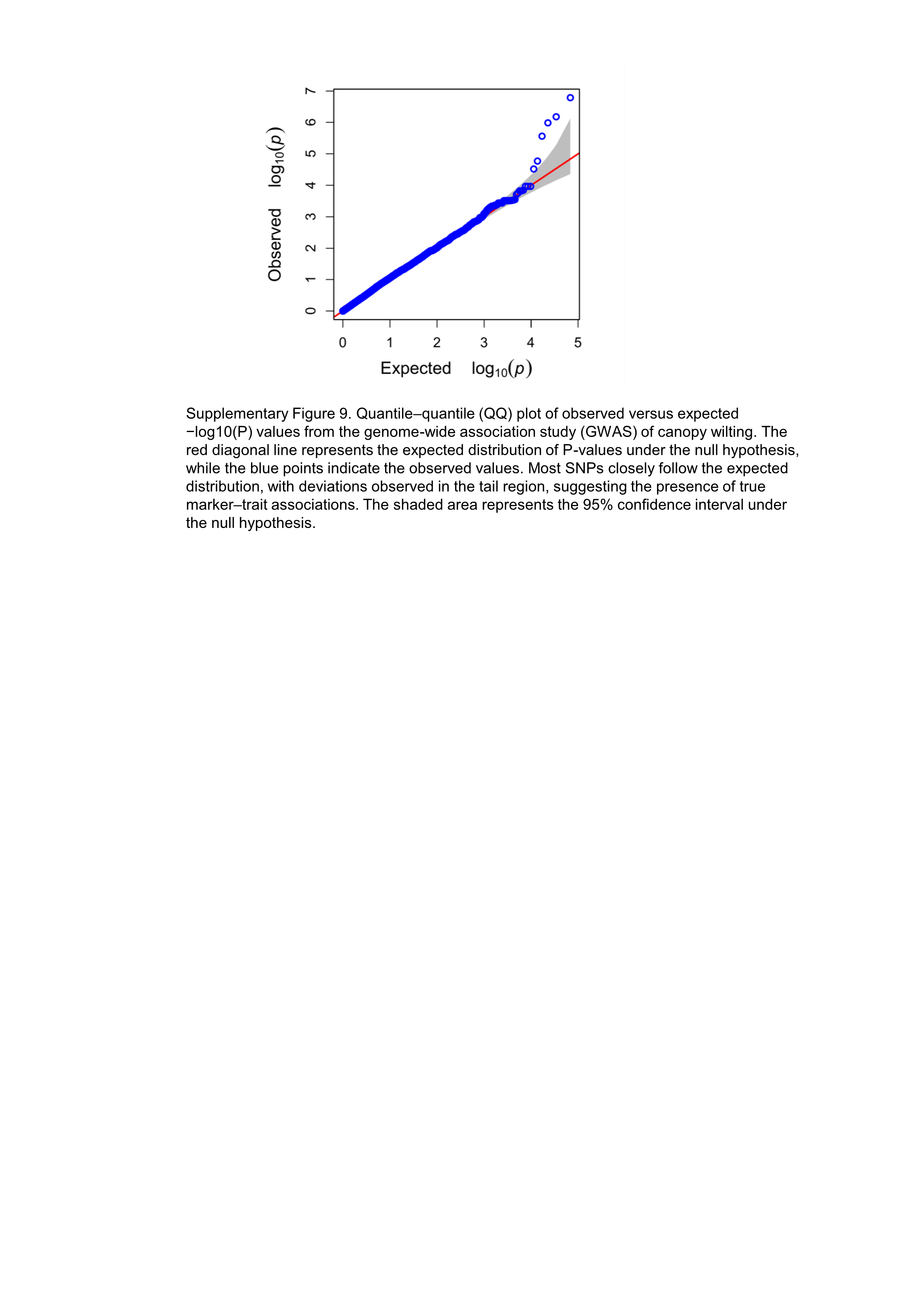

Supplement: Supplementary file 9 [file Image9.tif]
